# Global surgery teaching content in German universities: a mixed-method descriptive study

**DOI:** 10.1186/s12909-026-08856-x

**Published:** 2026-02-24

**Authors:** Linnea Weise, Cynthia Carvallo Fischer, Jarooshen Jayasingam, Judith Lindert

**Affiliations:** 1 Centre for Pediatric Surgery, University Hospital Giessen-Marburg, Marburg, Germany; 2 German Society of Global and Tropical Surgery e.V. DTC, Weimar, Germany; 3 Department of General and Visceral Surgery, Charité – University Hospital Charité, Berlin, Germany; 4 Sektion Kinderchirurgie, Universitätsklinikum Bonn, Bonn, Germany; 5 Department of Pediatric Surgery, University Medicine Rostock, Rostock, Germany

**Keywords:** Global Surgery, Global Health, Global Medicine, Education, Training, Germany, Electives

## Abstract

**Background:**

Despite increasing awareness of Global Surgery for surgeons worldwide for collaboration and exchange of resources with the final goal of making essential surgery available to all, only a few universities have included Global Surgery content in medical education. Ten years after the Lancet Commission report for Global Surgery, this descriptive study aimed to clarify the current status of teaching content on Global Surgery in German universities.

**Methods:**

In a mixed-method approach, all medical faculties in Germany were evaluated via a survey and available information on existing courses to clarify the teaching content of Global Surgery in Global Health-related programs. Practical training was highlighted. Statistical analysis and comparative studies using the Pearson chi-square test between types of programs, regions, and universities were performed.

**Results:**

Of the 45 universities, 36 (80%) offered one or several programs on Global Health, resulting in 81 programs, with the majority being Electives (*n* = 54; 66%). Global surgery content was included in seventeen universities (37%), including twenty-eight programs (34%), whereas four (14%) contained practical training. Educational hubs for Global Surgery seem to be Munich, Berlin, and Bonn. The reasons for the limited integration of Global Surgery in medical education are the current focus of programs and the lack of expertise and personnel.

**Conclusion:**

Most universities have incorporated Global Health into German medical education, but Global Surgery remains insufficiently represented, with limited teaching, scarce practical training, and fragmented institutional structures. Coordinated national strategies are needed to strengthen faculty capacity, integrate Global Surgery into existing curricula, and expand collaborative opportunities to prepare future health-care professionals to address global surgical inequities.

**Supplementary Information:**

The online version contains supplementary material available at 10.1186/s12909-026-08856-x.

## Background

Global Surgery, defined as “the multidisciplinary pursuit of improving health outcomes and achieving health equity for all people who need surgical care”, has become increasingly important for surgeons globally for collaboration and exchange of resources, with the final goal of making essential surgery available to all regardless of age, sex, race, ethnic group, geographical location, financial status, and political and religious affiliation [1]. The foundation of the Lancet Commission for Global Surgery (LCoGS) and the introduction of six core indicators to monitor national surgical system performance, as well as the World Health Assembly (WHA) Resolution 68.15, highlighted the need for action to strengthen the surgical capacity across all health care systems to reach that final goal [1, 2].

Despite increasing awareness of surgical inequity, Global Surgery as a topic remains insufficiently integrated into medical education, particularly in Germany [3–6]. Surveys of medical students demonstrate a strong interest in Global Surgery, yet exposure to structured teaching, electives, or clinical experiences remains highly variable and often absent [7–10]. Comparative analyses further show that while some universities have begun to incorporate Global Surgery through electives, online modules, or global health degrees, most medical curricula do not systematically address global surgical disparities, health-systems themes, or principles of global collaboration [3, 11–13].

Ten years after the landmark report by the LCoGS and the WHA Resolution 68.15 and given Germany’s growing engagement in global health, a systematic understanding of Global Surgery as a teaching content within German medical faculties is both timely and necessary [1, 2]. Documenting the current landscape provides a foundation for setting educational priorities, aligning with international recommendations, and preparing future clinicians to contribute to global surgical equity [5, 12, 14].

## Methods

With this descriptive study, we aimed to clarify the current status of Global Surgery content at medical universities for medical students and doctors in Germany. All officially registered universities in Germany with a medical faculty as of January 2025 were included. Universities without medical faculty were excluded. Private universities with medical faculties were included. A structured data collection tool was developed to systematically identify Global Health– and Global Surgery–related educational offerings. The tool captured predefined variables, including university name, course availability, course title, course type (e.g. elective, Master’s, Bachelor’s), level of study, course duration, and the availability of publicly accessible course descriptions.

Data were collected betwenn 1/25 − 10/25 through structured internet searches using Google Search and Ecosia Search. Searches combined each university name with predefined terms (“Global Health course,” “Global Surgery course,” “Global Medicine,” “International Medicine,” “Tropical Medicine,” and “Elective”), followed by a review of the official websites of the respective medical faculties.

To enhance data accuracy and completeness, the collected information was verified through direct email contact with each medical faculty using a short, standardized questionnaire. The questionnaire included the following questions:

- Does your program include a surgical lecture or a lecture on Global Surgery?
- Is there a practical component within the program related to Global Surgery?
- If yes, how long does the surgery-related component last?

**Fig. 1:**
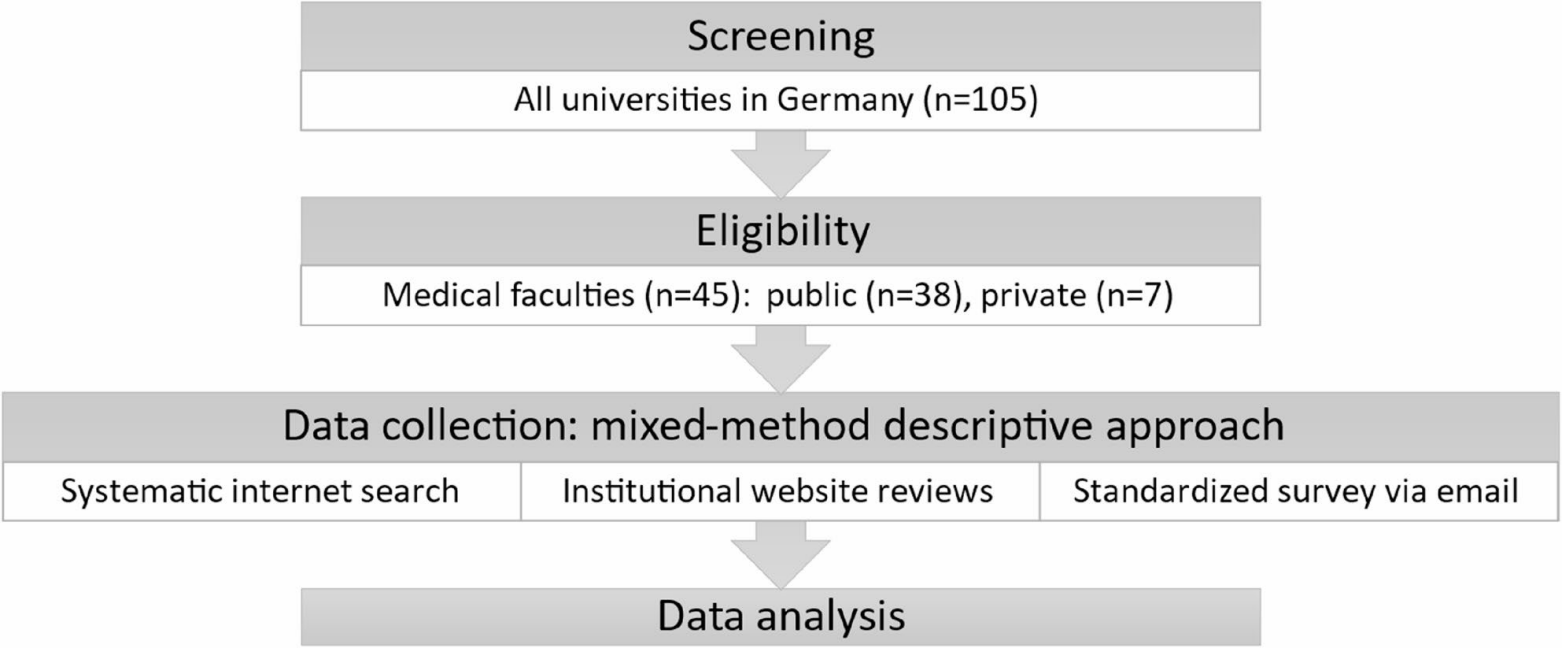
Flow diagram of the study methodology. All universities in Germany were screened for medical faculties, including all eligible institutions (*n* = 45), both public (*n* = 38) and private (*n* = 7) institutions. Data collection was performed using a mixed-method descriptive approach, as information was obtained through systematic internet searches, institutional website reviews, and direct email contact with medical faculties through a standardized survey

Figure 1 shows the flow diagram of the study methodology, classified as a mixed-method descriptive approach, as information was obtained through internet searches, institutional website reviews, and direct email contact with medical faculties to verify and complement the data.

All faculties were additionally asked to provide possible explanations for the absence or limited integration of Global Surgery content in their current medical education. The question was presented in a multiple-choice format with the option for elaboration. The respondents could select multiple reasons, including lack of relevance from the faculty’s perspective/different focuses or orientations, lack of staff or expertise, lack of financial resources, lack of interdisciplinary collaboration between Global Health and Global Surgery, lack of international exchange within the faculty, lack of student interest, or “other” with the option to provide comments.

The data were collected and analyzed via Microsoft Excel and the statistical software Jamovi Cloud [15, 16]. Statistical comparisons were performed via the Pearson chi-square test. A p-value < 0.05 was considered statistically significant. Artificial intelligence (ChatGPT by OpenAI) was used to assist with language refinement and editing during the preparation of the manuscript. Ethical clearance was obtained from the University of Rostock 2025 (A 2025-0049, 14.02.2025). The study was conducted in accordance with the Helsinki Guidelines.

## Results

### General results

**Fig. 2:**
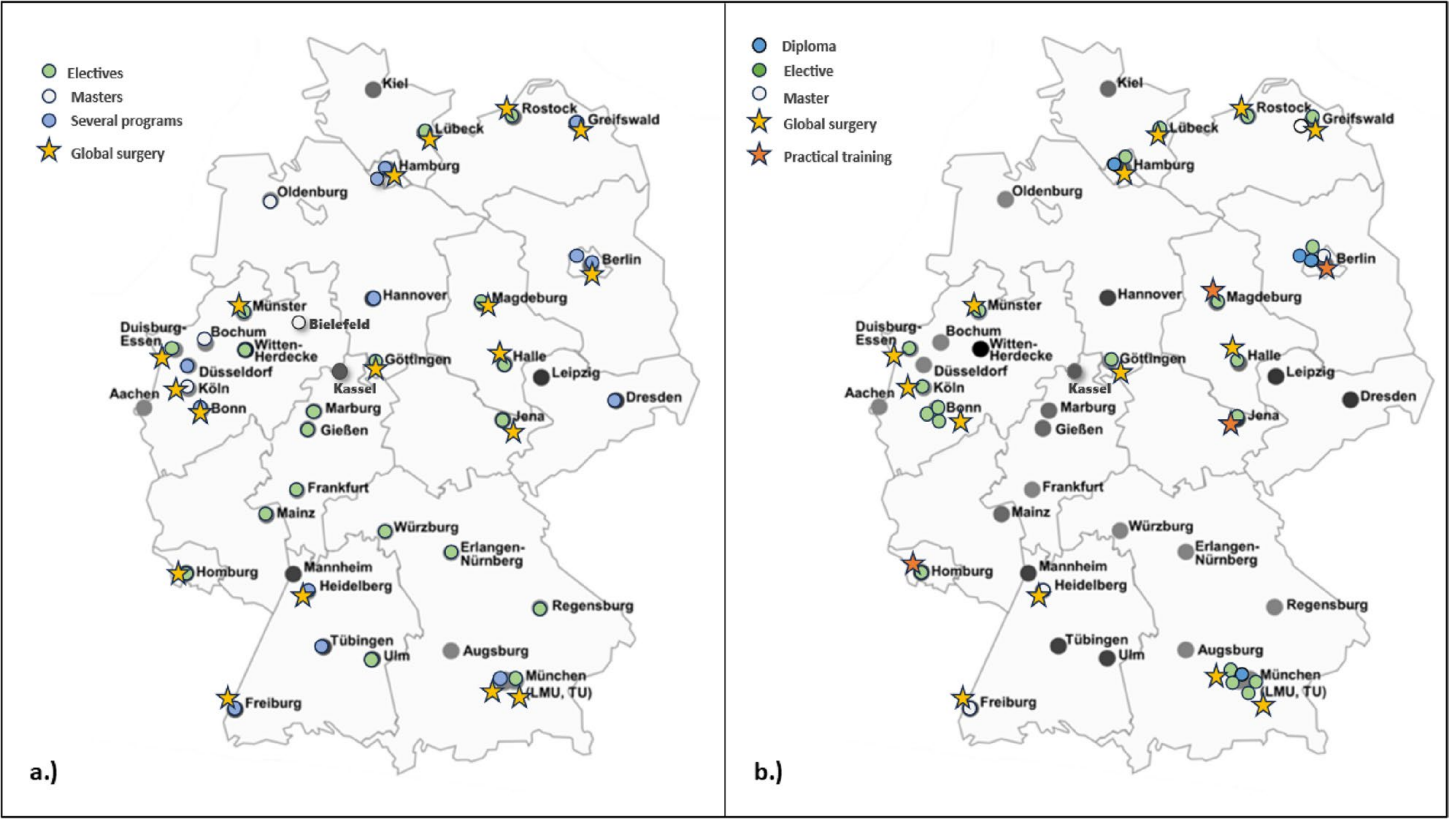
Map of Germany with all medical universities (**a**) Showing all faculties with programs on global health (*n* = 36; 80%), differentiated in types; green – only elective programs (*n* = 19; 42%), white – only master programs (*n* = 4; 11%); blue - more than one type of program (*n* = 13; 28%), star - including global surgery as a topic within the program (*n* = 18; 40%). **b **Highlights all courses on global health with global surgery content (*n* = 28; 34%), differentiated into types: green – elective (*n* = 20; 71%); white - master’s program (*n* = 3; 10%); blue - diploma program (*n* = 4; 14%); yellow star - global surgery (*n* = 24; 85%); and orange star – practical training (*n* = 4; 14%). Graphic has been modified. Copyright to Medical Education Research – Lehrforschung im Netz [17]

In total, 45 universities in Germany and their associated medical faculties were included in the study, seven (15%) of which were private universities. Among all the universities, 36 (80%) offered one or several programs on global health, resulting in the inclusion of 81 programs in total. Fifteen (33%) universities offered a master’s degree program, 32 (71%) universities offered an elective program during medical study, and thirteen (28%) universities offered more than one type of program. Table 1 provides an overview of the descriptive data of all the courses. Among all the programs on global health, the majority were electives (*n* = 54; 66%), followed by master’s programs (*n* = 19; 23%) and diploma courses (*n* = 4; 5%). The targeted audience was mainly medical students (*n* = 35; 43%). Global Surgery content was included in twenty-eight (34%) programs, whereas four (*n* = 4; 14%, *p* = 0.005) programs included practical surgical teaching as part of the global health program. Among all the universities, eighteen (40%) had one or more programs that included Global Surgery (Fig. 2). The duration of the teaching was mostly 90 min to three hours (*n* = 12; 42%). Among all the courses that included global surgery, the majority were elective (*n* = 20; 71.4%; *p* = 0.006), followed by master’s programs (*n* = 4; 14%). All four diploma courses included teaching content on global surgery (14.3%) (Table 1).

**Table 1:** Descriptive data of courses on Global Health in German universities

Descriptive data of the courses	
All programs on Global Health	
Master	19 (23%)
Elective	54 (66%)
Bachelor	3 (4%)
Diploma	4 (5%)
PHD	1 (1%)
Duration of the courses	
> 1 week	12 (14%)
1 week – 3 months	34 (42%)
3 months – 1 year	13 (16%)
> 1 year	22 (27%)
Target Audience	
Medical students	**35 (43%)**
Doctors	5 (6%)
Medical students & doctors	19 (23%)
All Health care professionals	22 (27%)
Global surgery/surgical lecture included, n (%)	28 (34%)
Type of programs with Global Surgery	
Master	4 (14%)
Elective	**20 (71%)**
Diploma	4 (5%)
Duration of surgery-related lecture	
< 90 min	9 (32%)
90 min–3 h	**12 (42%)**
3–6 h	2 (7%)
> 6 h	1 (4%)
Practical surgical teaching included, n (%)	4 (4%)

Displaying the types of all Global Health programs, the duration of the courses, the target audience, and the number of courses that include Global Surgery. The distribution of types of programs within the courses included Global Surgery, and the number of courses, including practical content. Bold: the majority within each category

## Regional comparison

**Fig. 3:**
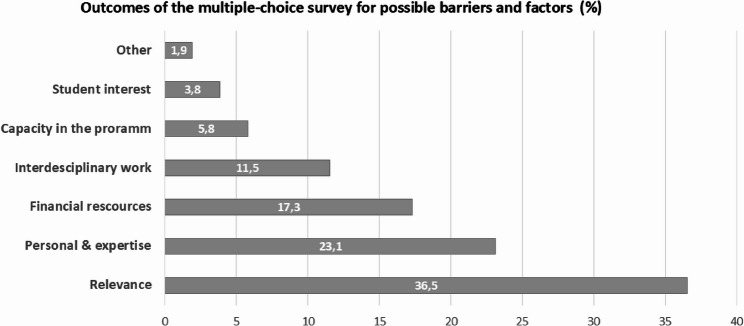
Bar chart: Outcome of the multiple-choice survey. Displaying possible barriers and explanations for the absence of Global Surgery content in the program, differentiated in the following: lack of relevance according to the program (*n* = 19; 36.5%), lack of personal and expertise (*n* = 11; 23.1%), lack of financial resources (*n* = 9; 17.3%), lack of interdisciplinary work (*n* = 6; 11.5%), lack of capacity in the program (*n* = 3; 5.8%), other reasons (*n* = 2; 3.8%), and lack of interest among students (*n* = 1; 1.9%)

Among all the regions, North Rhine-Westphalia offered the most courses on Global Health (*n* = 18; 22%, *p* = 0.926), followed by Bavaria (*n* = 15; 18%), Berlin (*n* = 11; 13.6%) and Baden-Württemberg (*n* = 9; 11%). Global surgery lectures were included mostly in North Rhine-Westphalia (*n* = 6; 21.4%, *p* = 0.150), followed by Bavaria (*n* = 5; 17.9%), Berlin (*n* = 4; 14%) and Mecklenburg-Vorpommern (*n* = 3; 10.7%). Controlled per university in each region, Berlin had the highest rate of courses per university, followed by Bavaria and Hamburg and Baden-Württemberg (Figs. 2 and 3). The general rate was 1.8 courses on Global Health per university in Germany.

### Comparing universities

Compared with all universities, Ludwig-Maximilians University (LMU) in Munich stands out, offering eight (9.9%, *p* = 0.999) courses on Global Health, followed by Charité University in Berlin (*n* = 5; 6.2%) and the University Witten/Herdecke (*n* = 4; 4.9%). The private university “Akkon Hochschule für Humanwissenschaften, Berlin” also offers six programs related to Global Health (7.4%). Global surgery lectures were included mostly at the University Charité Berlin (*n* = 4; 14.4%; *p* = 0.098), followed by Ludwig-Maximilians University in Munich (*n* = 3; 10.7%) and the University Bonn (*n* = 3; 10.7%).

### Survey for possible reasons

In total, 49 answers were obtained from the multiple-choice survey about possible reasons for the lack of Global Surgery content within the curricula. The majority (*n* = 19; 37%, *p* < 0.001), selected the lack of relevance of global surgery as a topic to the program as a reason. Further reasons were the lack of personnel and expertise (*n* = 11, 21%), the lack of financial resources (*n* = 9, 18%), the lack of interdisciplinary work (*n* = 6; 12%) and the lack of time capacity in the program (*n* = 3; 6%). One program was closed again because of the lack of interest from students (*n* = 1; 2%) (Fig. 3).

## Discussion

### Global Health and Global Surgery Education in Germany

This nationwide review revealed that ten years after the publication of the LCoGS, 80% of German medical faculties had incorporated at least one elective for medical students on Global Health; however, only 40% included any Global Surgery content, and practical surgical training was provided in four courses (Table 1) [1]. Most programs remain elective in nature, targeting medical students and embedding Global Surgery only marginally within broader Global Health curricula. Only the University of Bonn offers an elective exclusively dedicated to Global Surgery [18], whereas LMU Munich, Charité Berlin, and the University of Bonn appear to be educational hubs on Global Health (Fig. 2). These findings illustrate that Global Surgery is not systematically incorporated into medical education in Germany.

Our survey exploring explanations for the absence of Global Surgery content in German universities demonstrates that most Global Health programs prioritize other components of Global Medicine, such as infectious and neglected diseases, health policy, ethics, and health-care management, rather than Global Surgery. Limited curricular time was also cited as a barrier. The respondents further highlighted a shortage of academic expertise and personnel capable of delivering Global Surgery teaching, which additionally restricts its integration.

### Embedding Global Surgery education as a national strategy

These findings mirror earlier studies from Europe and North America, which report strong student interest in Global Surgery but limited structured educational opportunities and substantial variation across institutions [4, 7, 11, 14, 19, 20]. Reviews from the United Kingdom, the Netherlands, Belgium, and Scandinavia show that Global Surgery teaching often depends on motivated individual advocates rather than coordinated national strategies, resulting in fragmented and inconsistent curricula [3–5, 7, 11]. Our survey aligns with these observations, emphasizing that the lack of national coordination contributes substantially to the uneven integration of Global Surgery in Germany. In parallel with studies from the United Kingdom and the United States, our findings also show that some educators continue to associate Global Surgery primarily with humanitarian missions rather than with strengthening surgical systems, further marginalizing the field within academic curricula [9, 10, 21].

Although Global Surgery is closely connected to Global Health—and one might therefore question the need for stand-alone Global Surgery courses—the literature clearly underscores the importance of dedicated educational efforts. Reviews repeatedly demonstrate that medical students have limited knowledge of Global Surgery and are often dissatisfied with the opportunities available to them, despite strong overall interest. Student-led initiatives and international networks, such as InciSioN, have demonstrated that interest is not the limiting factor; instead, institutional support and faculty development remain key barriers across high-income countries [7–9].

### Distribution of Resources

The literature also highlights how this discrepancy can be addressed, offering a range of resources and examples for universities to use when establishing Global Surgery education. Rossi et al. identified twelve Global Surgery electives worldwide, of which four are in Europe, and proposed a structured model for undergraduate Global Surgery courses as a response to international variability [3]. In addition, research in Academic Global Surgery has defined essential competencies and learning objectives. Pawlak et al. outlined nine core competencies for Global Surgery education with learning objectives to compose and evaluate programs [22, 23], whereas Sherif et al. highlighted the ethical complexities that should be incorporated into teaching [24, 25]. Despite the availability of such resources, our study shows that only a minority of German universities have integrated these frameworks into their curricula, indicating a substantial gap between recommended educational practice and current implementation. Strengthening Global Surgery education may contribute to reducing global surgical inequities by developing a workforce that is aware of disparities in access to surgical care and trained in health systems–based approaches, as emphasized by the LCoGS [1].

Our survey on possible explanations for the scarcity of Global Surgery education highlights further key challenges that require attention. Faculties most frequently cited a perceived lack of relevance, shortages of academic expertise, and limited resources—including financial constraints—as major barriers to integrating Global Surgery into curricula. These findings are consistent with those of previous studies and reflect broader systemic issues. Similar factors influence postgraduate training pathways: Rosenberg et al. reviewed Global Surgery training opportunities for general surgeons and identified comparable barriers to engagement, such as lack of funding, time constraints, low faculty participation, and minimal institutional support [20]. Financial limitations appear to affect not only curricular development but also the feasibility of international rotations and the involvement of colleagues from low-income countries [13].

### Importance of International Rotations and Global Partnerships

While this study did not evaluate international rotations or university partnerships, the literature underscores their importance. Early and structured exposure during medical education enhances sustained engagement in Global Surgery, academic research, and ethical international collaborations, thereby supporting long-term capacity building and surgical system strengthening worldwide. Rotations for medical students and bilateral collaborations have been shown to strengthen academic Global Surgery, establish cooperation, and ultimately foster surgical equity worldwide [6, 12]. Exchange experiences have been associated with increased trainee interest in Global Surgery [26], and surveys of fellowship programs on Global Surgery by Davison et al. show a growing number of opportunities in the Global North, although there is a continuing need to strengthen collaboration with the Global South [14]. The willingness of surgeons from low-income countries to engage in academic partnerships [27] further suggests that gaps in expertise and resources could be reduced through structured collaborations.

### Need for Interdisciplinary and Longitudinal Approaches

Most programs in global health in Germany target only medical students or doctors, and most instructors are surgeons. However, Global Surgery inherently requires the involvement of diverse health-care professionals, policymakers, economists, and epidemiologists. This reflects findings from international reviews calling for broader interdisciplinary participation and a more equitable global distribution of educational opportunities [6, 28]. Evidence from other settings shows that sustained, longitudinal training, including health system modules, practical experiences, and supervised rotations, is more effective in building Global Surgery competencies than brief theoretical sessions alone [22, 24, 29] In line with LCoGS recommendations, early exposure during medical education is essential to cultivate a workforce capable of addressing global surgical challenges [3, 19].

### Limitations

This study has limitations, including reliance on publicly available information and variable survey response rates, which may underestimate existing but unpublished initiatives. Furthermore, the availability and usage of partnerships, exchange programs, and rotations were not specifically addressed. Nonetheless, the results clearly highlight opportunities for national coordination. Integrating Global Surgery into existing Global Health, public health, or surgical modules, supported by professional societies and academic networks, could help address the stated barriers.

## Conclusion

In summary, Global Surgery remains insufficiently represented in German medical education. Although most faculties offer Global Health electives, dedicated Global Surgery teaching and practical training are rare, largely because of limited resources, expertise, and institutional prioritization. These findings mirror international trends, where strong student interest contrasts with fragmented educational structures. There is a need for national and international strategies to address the fragmented state of Global Health and Global Surgery education, a task that may be particularly challenging in Germany because of the diversity of medical faculties and the independence of institutional educational frameworks.

Nevertheless, to better align with the aims of the Lancet Commission on Global Surgery, coordinated national efforts are needed to integrate Global Surgery more explicitly into existing curricula, strengthen institutional and faculty capacity, and expand collaborative opportunities. Ultimately, such measures will be essential to prepare future German physicians and health care professionals to engage effectively with global surgical inequities.

## Supplementary Information

Supplementary Material 1.

## Data Availability

Data generated or analyzed during this study are included in this published article and its supplementary information files. The contact details of the faculty are not publicly available to preserve privacy but are available from the corresponding author upon reasonable request.

## Abbreviations

LCoGS: Lancet Commission for Global Surgery
LMU: Ludwig-Maximilians University
WHA: World Health Assembly
PhD: Doctor of philosophy
