# Supplementary material for: Global surgery teaching content in German universities: a mixed-method descriptive study
Source: BMC Med Educ. 2026 Feb 24;26:362. doi: 10.1186/s12909-026-08856-x (PMC12955153; doi:10.1186/s12909-026-08856-x)
Supplement: Supplementary file 1 — Supplementary Material 1. [file 12909_2026_8856_MOESM1_ESM.docx]

**Additional file 1**

Table 1 Overview of Global Health courses per medical faculty

| **University** | **None** | **Master** | **Elective** | **Bachelor** | **pHD** | **Diploma** | **Total amounts of programs** | **Several programms** | **Global surgery content** | **Region** |
| --- | --- | --- | --- | --- | --- | --- | --- | --- | --- | --- |
| **Universität Freiburg** | 0 | 1 | 1 | 0 | 0 | 0 | 2 | 1 | 1 | BW |
| **Universität Heidelberg** | 0 | 1 | 2 | 0 | 0 | 0 | 3 | 1 | 1 | BW |
| **Universität Tübingen** | 0 | 1 | 2 | 0 | 0 | 0 | 3 | 1 | 0 | BW |
| **Ludwig-Maximilians-Universität München (LMU)** | 0 | 1 | 5 | 0 | 1 | 1 | 8 | 1 | 3 | BY |
| **Charité – Universitätsmedizin Berlin (gemeinsame Fakultät von FU Berlin und HU Berlin)** | 0 | 1 | 2 | 0 | 0 | 2 | 5 | 1 | 4 | BE |
| **Universität Hamburg (Medizinische Fakultät des UKE)** | 0 | 0 | 2 | 0 | 0 | 1 | 3 | 1 | 2 | HH |
| **Universität Greifswald** | 0 | 1 | 1 | 0 | 0 | 0 | 2 | 1 | 2 | MV |
| **Medizinische Hochschule Hannover (MHH)** | 0 | 2 | 1 | 0 | 0 | 0 | 3 | 1 | 0 | NI |
| **Universität Bonn** | 0 | 2 | 3 | 0 | 0 | 0 | 5 | 1 | 3 | NRW |
| **Universität Düsseldorf (HHU)** | 0 | 1 | 2 | 0 | 0 | 0 | 3 | 1 | 0 | NRW |
| **Technische Universität Dresden** | 0 | 1 | 1 | 0 | 0 | 0 | 2 | 1 | 0 | SN |
| **Akkon Hochschule für Humanwissenschaften (Berlin, Studiengang Public Health in Kooperation)** | 0 | 3 | 1 | 2 | 0 | 0 | 6 | 1 | 0 | BE |
| **Medical school Hamburg** | 0 | 1 | 0 | 1 | 0 | 0 | 2 | 1 | 0 | HH |
| **Total** | 9 | 19 | 54 | 3 | 1 | 4 | 81 | 13 | 28 |  |

*Showing all universities, showing the number of courses differentiated in master, elective, bachelor, diploma and pHD. Highlighting the number of courses with Global Surgery content. Results displayed with 0=no, 1= yes. Abbreviation: BW – Badenwürtenberg, BY – Baveria, BE – Berlin, HH – Hamburg, MV – Mecklenburg-Vorpommern, NI – Niedersachsen, NRW – Nordrheinwestfalen, SN – Sachsen-Anhalt*

Table 2 Results of the multiple-choice survey per region.

| **Possible barriers and factors** |  | **Baden-Württemberg** | **Bayern** | **Berlin** | **Bremen** | **Hamburg** | **Hessen** | **Mecklenburg-Vorpommern** | **Niedersachsen** | **Nordrhein-Westfalen** | **Rheinland-Pfalz** | **Sachsen** | **Sachsen-Anhalt** | **Schleswig-Holstein** | **Total** |
| --- | --- | --- | --- | --- | --- | --- | --- | --- | --- | --- | --- | --- | --- | --- | --- |
| **Relevance** | Observed | 5 | 3 | 0 | 1 | 0 | 0 | 1 | 3 | 5 | 0 | 1 | 0 | 0 | 19 |
|  | % within column | 50.0% | 42.9% | 0.0% | 100.0% | 0.0% | 0.0% | 50.0% | 100.0% | 26.3% | 0.0% | 50.0% | 0.0% | 0.0% | 36.5% |
| **Personal & Expertise** | Observed | 1 | 2 | 1 | 0 | 1 | 0 | 1 | 0 | 4 | 1 | 0 | 0 | 1 | 12 |
|  | % within column | 10.0% | 28.6% | 100.0% | 0.0% | 100.0% | 0.0% | 50.0% | 0.0% | 21.1% | 100.0% | 0.0% | 0.0% | 100.0% | 23.1% |
| **Financial resources** | Observed | 0 | 2 | 0 | 0 | 0 | 0 | 0 | 0 | 7 | 0 | 0 | 0 | 0 | 9 |
|  | % within column | 0.0% | 28.6% | 0.0% | 0.0% | 0.0% | 0.0% | 0.0% | 0.0% | 36.8% | 0.0% | 0.0% | 0.0% | 0.0% | 17.3% |
| **Interdesciplinary work** | Observed | 2 | 0 | 0 | 0 | 0 | 2 | 0 | 0 | 0 | 0 | 1 | 1 | 0 | 6 |
|  | % within column | 20.0% | 0.0% | 0.0% | 0.0% | 0.0% | 100.0% | 0.0% | 0.0% | 0.0% | 0.0% | 50.0% | 50.0% | 0.0% | 11.5% |
| **Time capacity** | Observed | 1 | 0 | 0 | 0 | 0 | 0 | 0 | 0 | 2 | 0 | 0 | 0 | 0 | 3 |
|  | % within column | 10.0% | 0.0% | 0.0% | 0.0% | 0.0% | 0.0% | 0.0% | 0.0% | 10.5% | 0.0% | 0.0% | 0.0% | 0.0% | 5.8% |
| **Interest of Students** | Observed | 1 | 0 | 0 | 0 | 0 | 0 | 0 | 0 | 0 | 0 | 0 | 0 | 0 | 1 |
|  | % within column | 10.0% | 0.0% | 0.0% | 0.0% | 0.0% | 0.0% | 0.0% | 0.0% | 0.0% | 0.0% | 0.0% | 0.0% | 0.0% | 1.9% |
| **Other** | Observed | 0 | 0 | 0 | 0 | 0 | 0 | 0 | 0 | 1 | 0 | 0 | 1 | 0 | 2 |
|  | % within column | 0.0% | 0.0% | 0.0% | 0.0% | 0.0% | 0.0% | 0.0% | 0.0% | 5.3% | 0.0% | 0.0% | 50.0% | 0.0% | 3.8% |
| **Total** | Observed | 10 | 7 | 1 | 1 | 1 | 2 | 2 | 3 | 19 | 1 | 2 | 2 | 1 | 52 |
|  | % within column | 100.0% | 100.0% | 100.0% | 100.0% | 100.0% | 100.0% | 100.0% | 100.0% | 100.0% | 100.0% | 100.0% | 100.0% | 100.0% | 100.0% |

*Displaying possible barriers and factors, differentiated in each region. Displaying the number of answers with percentages per collum*

Table 3 General data, showing all courses, classified for each university

| **ID** | **University** | **Region** | **Private** | **Type of course** | **Both Master + Elektive** | **None** | **Name of Course** | **Duration** | **Target Audience** | **Qualification** | **Global Surgery/Surgical lecture** | **Practical surgerical teaching** | **Duration of surgery-related lecture** | **Possible reason / Lack** |
| --- | --- | --- | --- | --- | --- | --- | --- | --- | --- | --- | --- | --- | --- | --- |
| 1.1 | Universität Freiburg | Baden-Württemberg | no | master | yes | no | Global Urban Health | n.a | All HCPs | master | yes | no | < 90min | Relevance |
| 1.2 | Universität Freiburg | Baden-Württemberg | no | elective | yes | no | Global Health | 1 week - 3 months | med. Students | elective | no | no | n.a. | time capacity |
| 2.1 | Universität Heidelberg | Baden-Württemberg | no | master | yes | no | Master of Science in International Health (MScIH) | 3 months - 1 year | All HCPs | master | yes | no | < 90min | Relevance |
| 2.2 | Universität Heidelberg | Baden-Württemberg | no | elective | yes | no | Global health wahlfachtrack | 3 months - 1 year | med. Students & doctors | certificate | no | no | n.a. | n.a. |
| 2.3 | Universität Heidelberg | Baden-Württemberg | no | elective | yes | no | Short coures | >1 week | med. Students & doctors | certificate | no | no | n.a. | Relevance |
| 3.1 | Universität Tübingen | Baden-Württemberg | no | elective | no | no | Blockkurs Globale Gesundheit mit Schwerpunkt Tropenmedizin, Reisemedizin und Humanparasitologie (Wahlfach) | 1 week - 3 months | med. Students | elective | no | no | n.a. | interdiciplinary work GH/GS |
| 3.2 | Universität Tübingen | Baden-Württemberg | no | elective | no | no | Global Health | 1 week - 3 months | med. Students | elective | no | no | n.a. | Students interest |
| 3.3 | Universität Tübingen | Baden-Württemberg | no | master | yes | no | Population-Based Medicine - Master | > 1 year | med. Students & doctors | master | no | no | n.a. | Relevance |
| 4.1 | Universität Ulm | Baden-Württemberg | no | elective | no | no | Global Health | 3 months - 1 year | med. Students | elective | no | no | n.a. | Relevance, personell, interdisciplinary work GH/GS |
| 5.1 | Ludwig-Maximilians-Universität München (LMU) | Bayern | no | master | yes | no | International Health | > 1 year | doctors | master | no | no | n.a. | Relevance |
| 5.2 | Ludwig-Maximilians-Universität München (LMU) | Bayern | no | elective | yes | no | EUGLOH Certificate of Advanced Studies in Global Health | 3 months - 1 year | med. Students | certificate | no | no | n.a. | no response |
| 5.3 | Ludwig-Maximilians-Universität München (LMU) | Bayern | no | phD | yes | no | Ph.D. Program Medical Research - International Health | > 1 year | med. Students & doctors | phD | no | no | n.a. | Relevance |
| 5.4 | Ludwig-Maximilians-Universität München (LMU) | Bayern | no | diploma | yes | no | Diploma in Tropical Medicine and International Health (DTMIH) | 1 week - 3 months | med. Students & doctors | diploma | yes | no | n.a. | no response |
| 5.5 | Ludwig-Maximilians-Universität München (LMU) | Bayern | no | elective | yes | no | MeCuM-Kurs: Global Health | 1 week - 3 months | med. Students | elective | yes | no | 90min | no response |
| 5.6 | Ludwig-Maximilians-Universität München (LMU) | Bayern | no | elective | yes | no | MeCuM-Kurs: Praktische Tropenmedizin für Famulatur und PJ | >1 week | med. Students | certificate | no | no | n.a. | n.a. |
| 5.7 | Ludwig-Maximilians-Universität München (LMU) | Bayern | no | elective | yes | no | Tropenmedizin + Global Health | >1 week | All HCPs | elective | yes | no | 90min | no response |
| 5.8 | Ludwig-Maximilians-Universität München (LMU) | Bayern | no | elective | yes | no | Advanced Modules in International Health | 1 week - 3 months | med. Students & doctors | certificate | no | no | n.a. | no response |
| 6.1 | Technische Universität München (TUM) | Bayern | no | elective | no | no | Wahlfach: Tropenmedizin | 1 week - 3 months | doctors | elective | no | no | n.a. | n.a. |
| 6.2 | Technische Universität München (TUM) | Bayern | no | elective | no | no | Vorlesung Global Health MSc und BSc | >1 week | med. Students & doctors | master | yes | no | 3-6h | personelle +expertise & finance |
| 6.3 | Technische Universität München (TUM) | Bayern | no | elective | no | no | Wahlpflichtfach: Medizin in den Tropen und Globale Gesundheit" | 1 week - 3 months | med. Students | elective | yes | no | 3-6h | personell, finance |
| 7.1 | Universität Erlangen-Nürnberg | Bayern | no | elective | no | no | Global Health 5 Projekte | >1 week | med. Students | elective | no | no | n.a. | no response |
| 8.1 | Universität Regensburg | Bayern | no | elective | no | no | Global Health | 1 week - 3 months | med. Students & doctors | elective | no | no | n.a. | Relevance |
| 9.1 | Universität Würzburg | Bayern | no | elective | no | no | Schwerpunktcurriculum Global Health and Care | > 1 year | med. Students | certificate | no | no | n.a. | no response |
| 9.2 | Universität Würzburg | Bayern | no | elective | no | no | Wahlpflichtfach: Global Health | 1 week - 3 months | med. Students | elective | no | no | n.a. | no response |
| 10.1 | Charité – Universitätsmedizin Berlin (gemeinsame Fakultät von FU Berlin und HU Berlin) | Berlin | no | master | yes | no | MSc International Health | > 1 year | med. Students & doctors | master | yes | no | 90min | personelle +expertise |
| 10.2 | Charité – Universitätsmedizin Berlin (gemeinsame Fakultät von FU Berlin und HU Berlin) | Berlin | no | diploma | yes | no | Diploma International Health and Tropical Medicine (Zusatzbezeichnung) | 3 months - 1 year | doctors | diploma | yes | no | 90min | n.a. |
| 10.3 | Charité – Universitätsmedizin Berlin (gemeinsame Fakultät von FU Berlin und HU Berlin) | Berlin | no | diploma | yes | no | Diploma International Public Health | 3 months - 1 year | All HCPs | diploma | yes | no | n.a. | n.a. |
| 10.4 | Charité – Universitätsmedizin Berlin (gemeinsame Fakultät von FU Berlin und HU Berlin) | Berlin | no | elective | yes | no | Einsatz und Katastrophenmedizin | 1 week - 3 months | med. Students | elective | yes | yes | > 6h | n.a. |
| 10.5 | Charité – Universitätsmedizin Berlin (gemeinsame Fakultät von FU Berlin und HU Berlin) | Berlin | no | elective | yes | no | Tropenmedizin und Internationale Gesundheit | 1 week - 3 months | med. Students | elective | no | no | n.a. | n.a. |
| 11.1 | Universität Bremen (Kooperationsstudiengang mit der Universität Oldenburg) | Bremen | no | master | no | no | Master Public Health - Gesundheitsförderung & Prävention | > 1 year | med. Students & doctors | master | no | no | n.a. | Relevance |
| 12.1 | Universität Hamburg (Medizinische Fakultät des UKE) | Hamburg | no | elective | no | no | Intermed - Globale Gesundheit, Kulturelle Kompentenz und Internationale Medizin | >1 week | med. Students & doctors | elective | no | no | n.a. | personelle +expertise |
| 12.2 | Universität Hamburg (Medizinische Fakultät des UKE) | Hamburg | no | elective | no | no | Tropenmedizin + Global Health | 1 week - 3 months | med. Students & doctors | certificate | yes | no | 90min | no response |
| 12.3 | Universität Hamburg (Medizinische Fakultät des UKE) | Hamburg | no | diploma | no | no | Diploma Tropical Medicine (Bernhard-Nocht-Institut für Tropenmedizin) | 3 months - 1 year | med. Students & doctors | diploma | yes | no | n.a. | n.a. |
| 13.1 | Goethe-Universität Frankfurt am Main | Hessen | no | elective | no | no | Current Topics in Tropical Medicine and Public Heath | 1 week - 3 months | med. Students | elective | no | no | n.a. | no response |
| 14.1 | Philipps-Universität Marburg | Hessen | no | elective | no | no | Global Health für Kliniker | >1 week | med. Students | elective | no | no | n.a. | no response |
| 14.2 | Philipps-Universität Marburg | Hessen | no | elective | no | no | Global Health für Vorkliniker | >1 week | med. Students | elective | no | no | n.a. | no response |
| 15.1 | Justus-Liebig-Universität Gießen | Hessen | no | elective | no | no | Einführung in Global Health | 1 week - 3 months | med. Students | elective | no | no | n.a. | interdiciplinary work GH / GS |
| 15.2 | Justus-Liebig-Universität Gießen | Hessen | no | elective | no | no | Tropenmedizin und Internationale Gesundheit | 1 week - 3 months | med. Students | elective | no | no | n.a. | interdiciplinary work GH / GS |
| 16.1 | Universität Greifswald | Mecklenburg-Vorpommern | no | master | yes | no | Health Care Management: Vorlesung "International Health Care Management" (Lehrstuhl für ABWL und Gesundheitsmanagement) | > 1 year | All HCPs | master | yes | no | < 90min | Relevance |
| 16.2 | Universität Greifswald | Mecklenburg-Vorpommern | no | elective | yes | no | Global Health und Tropenmedizin | 1 week - 3 months | med. Students | elective | yes | no | n.a. | personelle +expertise |
| 17.1 | Universität Rostock | Mecklenburg-Vorpommern | no | elective | no | no | "Tropen und Reisemedizin, Infektiologie und Parasitologie" | 1 week - 3 months | All HCPs | elective | yes | no | 90min | n.a. |
| 18.1 | Medizinische Hochschule Hannover (MHH) | Niedersachsen | no | master | no | no | Master of Science Public Health - Population and Professions | > 1 year | All HCPs | master | no | no | n.a. | Relevance |
| 18.2 | Medizinische Hochschule Hannover (MHH) | Niedersachsen | no | master | yes | no | Master programme Infectious Diseases and One Health Human - Animal - Environment | > 1 year | All HCPs | master | no | no | n.a. | Relevance |
| 18.3 | Medizinische Hochschule Hannover (MHH) | Niedersachsen | no | elective | yes | no | Wahlfach: Medizin und Krieg | 1 week - 3 months | med. Students | elective | no | no | n.a. | no response |
| 19.1 | Universität Göttingen (UMG) | Niedersachsen | no | elective | no | no | Tropenmedizin + Global Health | 1 week - 3 months | med. Students & doctors | elective | yes | no | 90min | no response |
| 19.2 | Universität Göttingen (UMG) | Niedersachsen | no | elective | no | no | Introduction to Global Health | >1 week | med. Students | bachelor | no | no | n.a. | Relevance |
| 20.1 | Universität Oldenburg (European Medical School, Kooperation mit Groningen, Niederlande) | Niedersachsen | no | none | no | yes | No Elective | n.a | n.a. | n.a. | no | no | n.a. | n.a. |
| 21.1 | RWTH Aachen | Nordrhein-Westfalen | no | none | no | yes | Global Health ? | n.a | n.a. | n.a. | no | no | n.a. | no response |
| 22.1 | Universität Bielefeld (neue Fakultät, seit Wintersemester 2021/22) | Nordrhein-Westfalen | no | master | no | no | Module Global Health | 1 week - 3 months | med. Students & doctors | master | no | no | n.a. | Relevance |
| 23.1 | Ruhr-Universität Bochum | Nordrhein-Westfalen | no | master | no | no | International Humanitarian Action | > 1 year | doctors | master | no | no | n.a. | no response |
| 24.1 | Universität Bonn | Nordrhein-Westfalen | no | master | yes | no | Kastastrophenvorsorge und -management | > 1 year | All HCPs | master | no | no | n.a. | Relevance |
| 24.2 | Universität Bonn | Nordrhein-Westfalen | no | master | yes | no | Glboal Health | > 1 year | All HCPs | master | no | no | n.a. | Relevance |
| 24.3 | Universität Bonn | Nordrhein-Westfalen | no | elective | yes | no | Section: Global Surgery | 1 week - 3 months | med. Students & doctors | elective | yes | no | < 90min | financial resource |
| 24.4 | Universität Bonn | Nordrhein-Westfalen | no | elective | yes | no | Wahlfach: Einführung in Global Health | 3 months - 1 year | med. Students | elective | yes | no | < 90min | financial resource |
| 24.5 | Universität Bonn | Nordrhein-Westfalen | no | elective | yes | no | Wahlfach: Global Health | 3 months - 1 year | med. Students | elective | yes | no | < 90min | financial resource |
| 25.1 | Universität Düsseldorf (HHU) | Nordrhein-Westfalen | no | elective | yes | no | Tropenmedizin und Global Health | 1 week - 3 months | med. Students | elective | no | no | n.a. | time capacity |
| 25.2 | Universität Düsseldorf (HHU) | Nordrhein-Westfalen | no | elective | yes | no | Kind, Gesundheit, Gesellschaft: Child Public Health and Health Services Research | 1 week - 3 months | med. Students | elective | no | no | n.a. | Relevance |
| 25.3 | Universität Düsseldorf (HHU) | Nordrhein-Westfalen | no | master | yes | no | Master: Public Health | > 1 year | All HCPs | master | no | no | n.a. | Relevance + Others |
| 26.1 | Universität Duisburg-Essen | Nordrhein-Westfalen | no | elective | no | no | Tropenmedizin & Global Health | 1 week - 3 months | med. Students & doctors | certificate | yes | no | 90min | n.a. |
| 27.1 | Universität Köln | Nordrhein-Westfalen | no | elective | no | no | Global Surgery | >1 week | doctors | elective | yes | no | < 90min | n.a. |
| 28.1 | Universität Münster | Nordrhein-Westfalen | no | elective | no | no | Internationale Kindergesundheit | 3 months - 1 year | med. Students | elective | yes | no | < 90min | n.a. |
| 28.2 | Universität Münster | Nordrhein-Westfalen | no | elective | no | no | Global Health | 1 week - 3 months | med. Students | elective | no | no | n.a. | time capacity |
| 29.1 | Johannes Gutenberg-Universität Mainz | Rheinland-Pfalz | no | elective | no | no | Internationale Gesundheit: Reise- und Tropenmedizin | 3 months - 1 year | med. Students | elective | no | no | n.a. | personelle +expertise |
| 30.1 | Universität des Saarlandes (Homburg/Saar) | Saarland | no | elective | no | no | Global Health | 1 week - 3 months | med. Students | elective | no | no | n.a. | n.a. |
| 30.2 | Universität des Saarlandes (Homburg/Saar) | Saarland | no | elective | no | no | Tropenmedizin und Global Health | 1 week - 3 months | med. Students & doctors | elective | yes | yes | 90min | n.a. |
| 31.1 | Universität Leipzig | Sachsen | no | none | no | yes | Global Health | n.a | n.a. | n.a. | no | no | n.a. | n.a. |
| 32.1 | Technische Universität Dresden | Sachsen | no | elective | yes | no | Global Health Initiative Spring School | >1 week | med. Students | certificate | no | no | n.a. | interdiciplinary work GH / GS |
| 32.2 | Technische Universität Dresden | Sachsen | no | master | yes | no | Public Health | > 1 year | All HCPs | master | no | no | n.a. | Relevance |
| 33.1 | Martin-Luther-Universität Halle-Wittenberg | Sachsen-Anhalt | no | elective | no | no | Globale und Planetare Gesundheit | 3 months - 1 year | med. Students | elective | yes | no | < 90min | interdisciplinary work GH/GS, Others |
| 34.1 | Otto-von-Guericke-Universität Magdeburg | Sachsen-Anhalt | no | elective | no | no | Tropenmedizin und Global Health | 1 week - 3 months | med. Students | elective | yes | yes | 90min | n.a. |
| 35.1 | Universität Kiel | Schleswig-Holstein | no | none | no | yes | Globale Medizin - One Health | n.a | med. Students | n.a. | no | no | n.a. | personelle +expertise |
| 36.1 | Universität Lübeck | Schleswig-Holstein | no | elective | no | no | Tropenmedizin + Global Health | 1 week - 3 months | med. Students & doctors | elective | yes | no | 90min | no response |
| 37.1 | Friedrich-Schiller-Universität Jena | Thüringen | no | elective | no | no | Tropenkurs Jena | 1 week - 3 months | med. Students | certificate | yes | yes | 90min | n.a. |
| 38.1 | Universität Witten/Herdecke | Nordrhein-Westfalen | yes | elective | no | no | Equity and child health rights | 1 week - 3 months | med. Students | certificate | no | no | n.a. | personelle +expertise & finance |
| 38.2 | Universität Witten/Herdecke | Nordrhein-Westfalen | yes | elective | no | no | Policy and Practice of Global Child Health | 1 week - 3 months | med. Students | certificate | no | no | n.a. | personelle +expertise & finance |
| 38.3 | Universität Witten/Herdecke | Nordrhein-Westfalen | yes | elective | no | no | Non-physician health care workers: leading change in global health | 1 week - 3 months | All HCPs | certificate | no | no | n.a. | personelle +expertise & finance |
| 38.4 | Universität Witten/Herdecke | Nordrhein-Westfalen | yes | elective | no | no | Summerschool Einführung in die Globale Kindergesundheit | >1 week | All HCPs | certificate | no | no | n.a. | personelle +expertise & finance |
| 39.1 | Akkon Hochschule für Humanwissenschaften (Berlin, Studiengang Public Health in Kooperation) | Berlin | yes | bachelor | no | no | Internationale Not- und Katastrophenhilfe B.A. | > 1 year | All HCPs | bachelor | no | no | n.a. | no response |
| 39.2 | Akkon Hochschule für Humanwissenschaften (Berlin) | Berlin | yes | bachelor | no | no | Management in der Gefahrenabwehr B.Sc. | > 1 year | All HCPs | bachelor | no | no | n.a. | no response |
| 39.3 | Akkon Hochschule für Humanwissenschaften (Berlin) | Berlin | yes | master | no | no | Global Health M.Sc. | > 1 year | All HCPs | master | no | no | n.a. | no response |
| 39.4 | Akkon Hochschule für Humanwissenschaften (Berlin) | Berlin | yes | master | no | no | Führung in der Gefahrenabwehr und im Krisenmanagement M.Sc. | > 1 year | All HCPs | master | no | no | n.a. | no response |
| 39.5 | Akkon Hochschule für Humanwissenschaften (Berlin) | Berlin | yes | master | no | no | Krisen-, Konflikt- und Katastrophenkommunikation M.A. | > 1 year | All HCPs | master | no | no | n.a. | no response |
| 39.6 | Akkon Hochschule für Humanwissenschaften (Berlin) | Berlin | yes | elective | yes | no | Weiterbildungsangebote aus dem Fachbereich Humanitäre Hilfe und Bevölkerungsschutz | > 1 year | All HCPs | certificate | no | no | n.a. | no response |
| 40.1 | Medizinische Hochschule Brandenburg Theodor Fontane (MHB) | Brandenburg | yes | none | no | yes | No Elective | n.a | n.a. | n.a. | no | no | n.a. | no response |
| 41.1 | Health and Medical University Potsdam (HMU) | Mecklenburg-Vorpommern | yes | none | no | yes | No Elective | n.a | n.a. | n.a. | no | no | n.a. | no response |
| 42.1 | Kassel School of Medicine (Kooperation mit der University of Southampton, UK) | Hessen | yes | none | no | yes | No Elective | n.a | n.a. | n.a. | no | no | n.a. | no response |
| 43.1 | Medical school Hamburg | Hamburg | yes | master | no | no | Notfall- und Krisenmanagement (M.Sc.) | > 1 year | All HCPs | master | no | no | n.a. | no response |
| 43.2 | Medical school Hamburg | Hamburg | yes | bachelor | no | no | Rescue Management (B.Sc.) | > 1 year | All HCPs | bachelor | no | no | n.a. | no response |
| 44.1 | Medical school Berlin | Berlin | yes | none | no | yes | No Elective | n.a | n.a. | n.a. | no | no | n.a. | no response |
| 45.1 | Universität Augsburg | Bayern | no | none | no | yes | No Elective | n.a | n.a. | n.a. | no | no | n.a. | n.a. |

*The table showing all results of the study, excluding contact-details. ID – course identification number, university, region – mentioned in German, private universities, type of course – differentiated into master, elective, bachelor, diploma and pHD, names of the course, the duration of the course, the target audience, the qualification achieved with participating the course, global surgery training included, practical surgery training included and results of the survey for possible reasons of the lack of global surgery content – differentiated in n.a. – not applicable, “no response”, “ack of finances”, “lack of personnel and expertise”, “lack of students interest”, “lack of interdisciplinary work between Global Surgery and Global Health”, “lack of time capacity”, “lack of relevance according to the faculty / programs goal”*
